# Reduced Regional Cerebral Blood Flow Relates to Poorer Cognition in Older Adults With Type 2 Diabetes

**DOI:** 10.3389/fnagi.2018.00270

**Published:** 2018-09-10

**Authors:** Katherine J. Bangen, Madeleine L. Werhane, Alexandra J. Weigand, Emily C. Edmonds, Lisa Delano-Wood, Kelsey R. Thomas, Daniel A. Nation, Nicole D. Evangelista, Alexandra L. Clark, Thomas T. Liu, Mark W. Bondi

**Affiliations:** ^1^Research Service, VA San Diego Healthcare System, San Diego, CA, United States; ^2^Department of Psychiatry, University of California, San Diego, San Diego, CA, United States; ^3^Department of Psychology, San Diego State University, San Diego, CA, United States; ^4^Psychology Service, VA San Diego Healthcare System, San Diego, CA, United States; ^5^Department of Psychology, University of Southern California, Los Angeles, CA, United States; ^6^Department of Radiology and Bioengineering, University of California, San Diego, San Diego, CA, United States

**Keywords:** aging, diabetes, vascular risk, arterial spin labeling, cerebral blood flow, neuropsychology, memory, Alzheimer’s disease

## Abstract

Type 2 diabetes mellitus (T2DM) increases risk for dementia, including Alzheimer’s disease (AD). Many previous studies of brain changes underlying cognitive impairment in T2DM have applied conventional structural magnetic resonance imaging (MRI) to detect macrostructural changes associated with cerebrovascular disease such as white matter hyperintensities or infarcts. However, such pathology likely reflects end-stage manifestations of chronic decrements in cerebral blood flow (CBF). MRI techniques that measure CBF may (1) elucidate mechanisms that precede irreversible parenchymal damage and (2) serve as a marker of risk for cognitive decline. CBF measured with arterial spin labeling (ASL) MRI may be a useful marker of perfusion deficits in T2DM and related conditions. We examined associations among T2DM, CBF, and cognition in a sample of 49 well-characterized nondemented older adults. Along with a standard T1-weighted scan, a pseudocontinuous ASL sequence optimized for older adults (by increasing post-labeling delays to allow more time for the blood to reach brain tissue) was obtained on a 3T GE scanner to measure regional CBF in FreeSurfer derived regions of interest. Participants also completed a neuropsychological assessment. Results showed no significant differences between individuals with and without T2DM in terms of cortical thickness or regional brain volume. However, adjusting for age, sex, comorbid vascular risk factors, and reference CBF (postcentral gyrus) older adults with T2DM demonstrated reduced CBF in the hippocampus, and inferior temporal, inferior parietal, and frontal cortices. Lower CBF was associated with poorer memory and executive function/processing speed. When adjusting for diabetes, the significant associations between lower regional CBF and poorer executive function/processing speed remained. Results demonstrate that CBF is reduced in older adults with T2DM, and suggest that CBF alterations likely precede volumetric changes. Notably, relative to nondiabetic control participants, those with T2DM showed lower CBF in predilection sites for AD pathology (medial temporal lobe and inferior parietal regions). Findings augment recent research suggesting that perfusion deficits may underlie cognitive decrements frequently observed among older adults with T2DM. Results also suggest that CBF measured with ASL MRI may reflect an early and important marker of risk of cognitive impairment in T2DM and related conditions.

## Introduction

Type 2 diabetes mellitus (T2DM) is a chronic, highly disabling metabolic disorder that is growing in prevalence at an alarming rate. In 2015, it was estimated that 30.3 million Americans (1 in 10) had either diagnosed or undiagnosed diabetes, the vast majority of whom (90–95%) had T2DM (Prevention, 2017). This is particularly concerning, given that T2DM has been linked to an increased risk for developing mild cognitive impairment (MCI) and dementia including Alzheimer’s disease (AD) (Luchsinger et al., 2001; Arvanitakis et al., 2004; Luchsinger et al., 2007; Vagelatos and Eslick, 2013) – conditions that are associated with high health care costs, reduced quality of life, and loss of independence in late adulthood. Even among nondemented older adults, studies have observed increased rates of subtle cognitive impairment and accelerated cognitive decline in individuals with T2DM compared to their nondiabetic counterparts, suggesting that cognitive impairment may be a chronic long-term complication of T2DM (Geijselaers et al., 2015). In T2DM, vascular dysfunction has long been considered the underlying cause of the multi-organ complications of the disease. Cerebrovascular dysfunction, thus, seems to be a possible mechanism by which poor cognitive outcomes occur in diabetes. Indeed, neuropathologic studies have linked T2DM to increased incidence of cerebral infarcts (Peila et al., 2002; Arvanitakis et al., 2006; Sarwar et al., 2010). Furthermore, insulin resistance, hyperglycemia, and inflammation – all which represent defining deleterious metabolic states that occur in diabetes – have been linked to cerebrovascular dysfunction (Brownlee, 2005; Zhou et al., 2014; Chung et al., 2015).

Most previous studies of brain changes underlying cognitive decrements in T2DM have applied conventional structural magnetic resonance imaging (MRI) to detect macrostructural changes associated with cerebral gray matter (GM) atrophy and markers of cerebrovascular disease (CVD) lesions such as white matter hyperintensities (WMH), which are thought to reflect small vessel disease. Many structural neuroimaging studies have shown *in vivo* cerebral atrophy in T2DM that has been linked to poorer cognitive performance across domains including memory, executive functioning, and processing speed (Tiehuis et al., 2009; Hayashi et al., 2011; Moran et al., 2013; Zhang et al., 2014). Results from several of these studies indicate that regional atrophy patterns in T2DM resemble those seen in preclinical AD, with hippocampal atrophy identified as the earliest and most prominent neurodegenerative change (Moran et al., 2013). However, these structural brain changes in T2DM likely reflect end-stage manifestations of chronic decrements in cerebrovascular functioning. Advanced functional MRI techniques that measure cerebral blood flow (CBF) may elucidate the mechanisms that precede the development of irreversible parenchymal damage and serve as an early indicator of impending cognitive decline in at-risk populations.

Arterial spin labeling (ASL) is a non-invasive MRI technique that measures CBF alterations. ASL studies of AD demonstrate similar patterns of regional perfusion compared to studies using fluorodeoxyglucose positron emission tomography (FDG-PET) and single photon emission computed tomography (SPECT) (Chen et al., 2011; Takahashi et al., 2014). ASL techniques have advantages over PET and SPECT, however, related to the nature of the tracer (i.e., magnetically labeled arterial water) (Detre and Alsop, 1999). That is, ASL employs a non-invasive, endogenous tracer rather than an intravenously administered contrast agent. The rapid decay time of the magnetized water molecules (on the order of seconds), moreover, allows for relatively brief scan times (5–10 min) that can provide dynamic CBF estimates with high temporal resolution (Johnson et al., 2005). These advantages, combined with its ability to quantitatively measure cerebral perfusion (in milliliters per 100 g of tissue per minute), make ASL an ideal technique for research and clinical settings (Telischak et al., 2015) designed to monitor neural and vascular changes in healthy older adults (Bangen et al., 2009) and clinical populations (Johnson et al., 2005; Xu et al., 2007; Bangen et al., 2012; Binnewijzend et al., 2013).

There are few publications examining cerebral perfusion and its associations with cognition in individuals with T2DM, and findings across these limited studies are contradictory. In one of the earliest studies, Dandona et al. (1978) measured global CBF by the 133-Xe inhalation method in 59 individuals with T2DM and 28 controls encompassing a wide range of ages. They reported age-related perfusion reductions that were similar in those with and without T2DM. Previous studies employing SPECT have reported that diabetic patients exhibit decreased CBF (Wakisaka et al., 1990; Nagamachi et al., 1994; Sabri et al., 2000) and that these CBF reductions are associated with poorer cognitive performance (Xia et al., 2015). Although the extant literature still remains relatively limited, a handful of studies have attempted to employ ASL to assess associations among diabetes status, CBF, and cognitive functioning. Consistent with the PET and SPECT literature, several studies have reported regional cerebral hypoperfusion in individuals with diabetes (Last et al., 2007; Xia et al., 2015; Cui et al., 2017; Dai et al., 2017), although some studies have reported no such differences between individuals with and without diabetes (Launer et al., 2015; Rusinek et al., 2015). Those studies that do report significant differences, however, have most consistently observed associations between diabetes status and hypoperfusion in posterior cortical regions (e.g., parietal regions) (Last et al., 2007; Xia et al., 2015; Cui et al., 2017; Dai et al., 2017), although some reports document hypoperfusion in frontal, temporal, and limbic regions as well (Xia et al., 2015; Cui et al., 2017; Dai et al., 2017). With respect to cognitive functioning in T2DM, the ASL literature is even further limited. While the available evidence suggests that alterations to CBF in certain regions are associated with poorer cognitive functioning in individuals with T2DM (Novak et al., 2014; Xia et al., 2015; Cui et al., 2017; Dai et al., 2017), these data are extremely limited given the very few published studies to date, and the findings are mixed with respect to the affected cognitive domains.

Although there is mounting evidence to suggest an association between T2DM, CBF, and cognition, prior studies lack the robust methodology needed to reliably assess these associations. Many studies do not specifically explore these associations in an older adult population, despite this population being at an elevated risk for both T2DM and dementia. Moreover, those that do target an older sample employ ASL methods that are not optimized for imaging CBF in older adults, which is problematic considering that this population has expected increases in transport time from the labeling position to the tissue (i.e., longer arterial transit time) relative to younger adults and therefore the post-labeling delay should be adjusted accordingly so that the CBF estimation will not be biased by incomplete delivery of the labeled bolus prior to image acquisition (Alsop et al., 2015). Finally, most studies include relatively limited neuropsychological assessment. Thus, the present study sought to extend the literature by examining the associations among T2DM, CBF, and cognition in a sample of well-characterized nondemented older adults who underwent pseudo-continuous ASL imaging optimized for older adult populations and comprehensive neuropsychological assessment.

## Materials and Methods

### Participants

Forty-nine independently living, nondemented older adults were recruited from ongoing aging studies at the University of California, San Diego (UCSD) and the San Diego VA Healthcare System. Potential participants were excluded if they were younger than 60 years of age; had a history of Type 1 diabetes; had dementia identified by medical, neurological, and neuropsychological examinations; or had a history of stroke or neurologic disease (e.g., Parkinson’s disease, multiple sclerosis), head injury with cognitive sequelae, or major psychiatric disorder; or for whom MRI was contraindicated (e.g., individuals with a pacemaker). Of the 49 participants, 11 had T2DM, and 38 were nondiabetic control participants.

### Ethics Statement

All participants provided written informed consent prior to enrollment, and data were collected in accordance with ethical standards for research. The UCSD and VA San Diego Healthcare System Institutional Review Boards approved the research protocol.

### Clinical and Neuropsychological Assessment

All participants underwent a semi-structured clinical interview assessing medical and psychiatric history; assessment of instrumental activities of daily living; physical examination with brachial artery blood pressure measurement using an automated blood pressure cuff; comprehensive neuropsychological testing; and brain MRI. Participants were classified as having diabetes based on self-report during clinical interview and review of available medical records. Of the 11 participants with T2DM, 10 were being treated with antidiabetic medications (9 with oral glucose lowering agents only and 1 with insulin only).

Presence of additional vascular risk factors included in the Framingham stroke risk profile (FSRP) (D’Agostino et al., 1994) was determined by self-report, medical chart review, and physical examination. These vascular risk factors included: (1) hypertension (defined as systolic blood pressure ≥140 mm Hg, diastolic blood pressure ≥90 mm Hg, or use of antihypertensive medications); (2) history of cardiovascular disease [e.g., coronary artery disease (myocardial infarction, angina pectoris, coronary insufficiency), intermittent claudication, cardiac failure]; (3) atrial fibrillation; and (4) current cigarette smoking. To characterize the aggregate vascular risk burden of our sample, we also calculated a modified FSRP for each participant (D’Agostino et al., 1994) omitting diabetes. Pulse pressure – a proxy for arterial stiffness – was also calculated (as systolic minus diastolic blood pressure), given that T2DM is associated with arterial stiffening (Winer and Sowers, 2003; Tomiyama et al., 2005). Arterial stiffening is also an AD risk factor that relates to AD cerebrospinal fluid (CSF) biomarkers and cerebrovascular functioning (Nation et al., 2015, 2016b; Werhane et al., 2018) including reduced CBF (Yan et al., 2016).

Global cognition was assessed by the dementia rating scale (DRS) (Mattis, 1988). Episodic memory was assessed by the California Verbal Learning Test-Second Edition (CVLT-II) (Delis et al., 2000) long delay free recall, and delayed recall of the Logical Memory and Visual Reproduction subtests of the Wechsler memory scale – revised (WMS-R) (Wechsler, 1987). Executive function/processing speed was assessed using Trail Making Test, Parts A and B. For each of the five cognitive scores, each participant’s raw score was converted to a *z*-score based on the mean and standard deviation of the entire sample (*n* = 49). Domain composite scores are the mean of *z*-scores measured within that domain. In addition, executive function/processing speed composite scores were multiplied by –1 so that positive *z*-scores represented better performance for all scores. Of note, one participant with T2DM was missing CVLT-II data; one nondiabetic control participant was missing WMS-R Logical Memory data; and one T2DM participant was missing WMS-R Visual Reproduction data. For these three participants who were each missing data for one memory measure, their memory composite score was calculated as the mean of their two existing memory scores. In addition, two T2DM participants were missing both Trail Making Test variables and, therefore, these individuals were not included in the analyses involving the executive function/processing speed composite.

### MRI Data Acquisition

Magnetic resonance imaging data were acquired on one of two identical GE Discovery MR 750 3T whole body systems using an 8-channel receive-only head coil (General Electric Medical Systems, Milwaukee, WI, United States) at the UCSD Keck Center for functional MRI. During scanning, participants are provided with ear plugs and MRI-safe noise reduction headphones and instructed to stay still. The scanner room is dark and there is no visual stimulation. Participants are not given instructions to keep their eyes open or closed. A T1-weighted anatomical scan was acquired using a Fast Spoiled Gradient Recall (3DFSPGR) sequence with the following parameters: 172.1 mm contiguous sagittal slices, field of view (FOV) = 25 cm, repetition time (TR) = 8 ms, echo time (TE) = 3.1 ms, flip angle = 12, inversion time (TI) = 600 ms, 256 × 192 matrix, bandwidth = 31.25 kHZ, frequency direction = S–I, NEX = 1, scan time = 8 min, and 13 s.

Resting CBF was acquired using a 2D pseudocontinuous ASL (PCASL) sequence optimized for older adult populations, which increases post-labeling delays to allow more time for the blood to reach brain tissue with the following parameters: TR = 4,500 ms, TE = 3.2 ms, FOV = 24 cm, labeling duration = 1,800 ms, post-labeling delay = 2,000 ms, 24.6 mm slices, with a single shot spiral acquisition and a total scan time of 4:18 min plus a 30 s calibration scan. In addition, a spiral scan with the inversion pulses turned off was acquired to obtain an estimate of the magnetization of CSF. The CSF signal from this scan was used to estimate the equilibrium magnetization of blood, which was used to convert the perfusion signal into calibrated CBF units (i.e., millimeters of blood per 100 g of tissue per minute) (Chalela et al., 2000). A minimum contrast scan was also acquired to adjust for coil inhomogeneities during the CBF quantification step (Wang et al., 2005). Finally, a field map scan was also acquired and used for off-line field map correction to help correct distortion and signal dropout, particularly in the frontal and medial temporal lobes.

### MRI Data Processing

MRI data were processed using Analysis of Functional NeuroImages (AFNI) (Cox, 1996), FMRIB Software Library (FSL) (Smith et al., 2004), FreeSurfer, and locally created Matlab scripts.

#### T1-Weighted Anatomical Images

T1-weighted anatomical images were processed using FreeSurfer 5.1 software. Briefly, images underwent skull stripping, B1 bias field correction, GM–WM segmentation, reconstruction of cortical surface models, and parcellation and labeling of regions on the cortical surface as well as segmentation and labeling of subcortical brain structures (Dale et al., 1999; Fischl et al., 2002). FreeSurfer output (gray–white boundary surface, pial surface, cortical parcellation, and subcortical segmentation) was visually inspected and, when necessary, manual edits were performed to ensure proper region of interest (ROI) segmentation and GM and WM differentiation.

#### ASL Images

Each participant’s raw ASL data (perfusion, CSF, and mincon data), field map, and anatomical data were uploaded for processing to the Cerebral Blood Flow Biomedical Informatics Research Network (CBFBIRN ^1^) (Shin et al., 2013) established at the UCSD Center for Functional Magnetic Resonance Imaging). Field map and motion correction; skull-stripping; and tissue segmentation using FSL’s Automated Segmentation Tool (FAST) algorithm to define CSF, GM, and WM tissue were completed. The high-resolution T1-weighted image and partial volume segmentations were then registered to ASL space, and partial volume segmentations were down-sampled to the resolution of the ASL data. To correct the CBF measures for partial volume effects and ensure that CBF values were not influenced by known decreased perfusion in WM or increased volume of CSF (Parkes et al., 2004), we utilized the method described by Johnson et al. (2005). These calculations assume that CSF has 0 CBF and that CBF in GM is 2.5 times higher than that in WM. To compute partial volume corrected CBF signal intensities, the following formula was used: CBFcorr = CBFuncorr/(GM + 0.4 × WM) where CBFcorr and CBFuncorr are corrected and uncorrected CBF values, respectively, and GM and WM are the partial volume fractions of GM and WM, respectively. The CBFcorr data was blurred to 4.0 mm full-width at half maximum. Each participant’s quantified CBF map (in units of mL/100 g tissue/min) was downloaded to a local server and a threshold was applied that removed values outside of the expected physiological range of CBF (<10 or >150) (Bangen et al., 2014).

**FIGURE 1:**
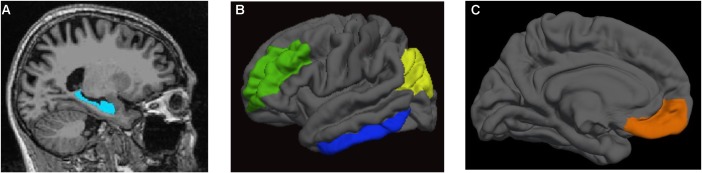
Regions of interest. **(A)** hippocampus; **(B)** rostral middle frontal gyrus (in *green*), inferior temporal cortex (in *blue*), and inferior parietal cortex (in *yellow*); **(C)** medial orbitofrontal cortex (in *orange*).

FreeSurfer was used to generate anatomical ROIs for the CBF data as well cortical thickness and volume data for these ROIs to be compared between the T2DM and nondiabetic control groups. We examined left and right hemisphere for the following five *a priori* ROIs: (1) hippocampus, (2) inferior temporal cortex, (3) inferior parietal cortex, (4) rostral middle frontal gyrus, and (5) medial orbitofrontal cortex. These ROIs were selected because they have been implicated in cerebrovascular dysfunction in MCI and AD (Du et al., 2002; Nation et al., 2013, 2016a). Many of these regions have been implicated in T2DM in the few existing studies of ASL CBF in this population (Last et al., 2007; Xia et al., 2015; Cui et al., 2017; Dai et al., 2017). The regional GM CBF values (corrected for partial volume effects) from the Desikan et al. (2006) atlas were extracted for each of the ROIs for each hemisphere. See **Figure 1** for a depiction of the *a priori* ROIs used in the primary analyses. In addition, to adjust for individual variation in CBF, postcentral gyrus CBF was used as a reference region and included as a covariate in statistical analyses comparing groups on CBF in the ROIs. This region was selected due to its relative sparing in AD (Thompson et al., 2003) and T2DM-related brain atrophy (Moran et al., 2013; Zhang et al., 2014) as well as its use as a control region in our prior studies of CBF in older adults at increased risk for AD (Bangen et al., 2017). FreeSurfer-derived intracranial volume was used as a covariate in analyses comparing groups on regional brain volume.

In addition, groups were also compared on FreeSurfer-derived volumes of WM signal abnormalities (WMSAs). WMSAs on MRI refer to regions in the WM that appear hyperintense on T2 fluid-attenuated inversion recovery (FLAIR) but hypointense on T1-weighted images. WMSAs are often observed in aging and conditions including diabetes and are usually thought to reflect small vessel CVD resulting from microvascular hypoperfusion (Garde et al., 2000; O’Sullivan et al., 2002; Brickman et al., 2015; Shen et al., 2017). The FreeSurfer automated segmentation pipeline subdivides brain tissue into regions of GM, WM, and hypointense regions within the WM using a combination of segmentation and a set of anatomical priors (Fischl and Dale, 2000; Fischl et al., 2002). Total volume of WM hypointensities was extracted from FreeSurfer output.

### Statistical Analyses

Analysis of variance (ANOVA) and chi-square tests were used to compare those with and without T2DM on demographic and clinical characteristics of interest. Multiple analysis of covariance (MANCOVA) models were used to determine CBF and structural brain differences between T2DM and nondiabetic control participants. The MANCOVA compared CBF in the selected ROIs adjusting for age, sex, modified FSRP (omitting diabetes), and reference CBF (postcentral gyrus). The modified FSRP was used as a covariate in an effort to determine whether any potential groups differences in CBF were related to T2DM rather than possible comorbid vascular risk factors/conditions. This composite measure to assess vascular risk (rather than individuals vascular risk factors) was used to maximize the sample size to independent variable ratio in our analyses. MANCOVAs were also used to determine whether there were group differences in brain structure (i.e., cortical thickness or volume) that might influence CBF findings, particularly given that some previous studies have reported that atrophy may largely explain lower CBF in T2DM (Sabri et al., 2000). One MANCOVA compared cortical thickness in 4 of the 5 ROIs (inferior parietal cortex, inferior parietal cortex, rostral middle frontal gyrus, and medial orbitofrontal cortex) adjusting for age, sex, and modified FSRP. A second MANCOVA compared volume of hippocampus and WM hypointensities adjusting for age, sex, modified FSRP, and intracranial volume. All *a priori* ROIs were entered into the MANCOVAs simultaneously.

Pearson’s product-moment correlations examined the associations between cognition and CBF across the entire sample (i.e., collapsed across T2DM and nondiabetic control participants). To minimize comparisons, we examined associations only for those unilateral ROIs that showed significant group differences in CBF. For each significant ROI, we correlated regional CBF with performance in cognitive domains subserved by that region. Specifically, we examined the associations of the memory composite score and CBF in the hippocampus and inferior temporal cortex. In addition, we examined the associations of the executive function/processing speed composite with CBF in the inferior parietal cortex and rostral middle frontal gyrus.

Sensitivity analyses were performed to determine whether results from the primary analyses may have been influenced by potential sex-related CBF differences and/or the presence of comorbid vascular risk factors associated with T2DM (e.g., hypertension). First, we compared men and women (regardless of T2DM status) on mean CBF for regions where differences were observed between the T2DM and nondiabetic control groups. Second, we ran *t*-test analyses to compare a subset of the sample consisting of nondiabetic control participants (*n* = 11) and T2DM (*n* = 11) participants who were matched so that the two groups did not significantly differ in terms of demographic or covariate variables. Significance levels of 0.05 were used for all analyses. All statistical analyses were conducted using the Statistical Package for the Social Sciences (SPSS) version 24 (SPSS IBM, New York, United States).

Finally, in order to address potential inflation of type I error resulting from multiple comparisons, we applied the Benjamini–Hochberg procedure (Benjamini and Hochberg, 1995). We assessed results when the false discovery rate (FDR) was controlled at 0.05 and 0.10.

## Results

### Participant Characteristics

Participant demographics and clinical characteristics are presented in **Table 1**. In comparison to the nondiabetic control group, the T2DM group had a greater proportion of men relative to women and a greater proportion of individuals with a history of hypertension relative to those with no history of hypertension. There were no significant group differences with respect to mean age, education, aggregate vascular risk (i.e., modified FSRP omitting diabetes), pulse pressure, current smoking status, history of cardiovascular disease, history of atrial fibrillation, and depression. There were no significant group differences in global cognition as assessed by DRS total score. In contrast, those with T2DM performed significantly worse on the memory composite score and the executive function/processing speed composite score.

**Table 1:** Demographic and neuropsychological characteristics by T2DM status.

	Nondiabetic (*n* = 38)	T2DM (*n* = 11)	*F* or *χ*^2^	*p*	Effect size
**Demographics^∗^**					
Age, years	73.6 (5.9)	72.3 (2.8)	0.49	0.486	${\text{η}}_{p}^{2}$ = 0.010
Education, years	17.0 (1.7)	16.6 (2.4)	0.24	0.627	${\text{η}}_{p}^{2}$ = 0.005
Sex, M:F	13:25	8:3	5.17	0.023	φ_c_ = 0.325
FSRP (%)^∗∗^	10.1 (7.9)	13.4 (7.8)	1.47	0.232	${\text{η}}_{p}^{2}$ = 0.030
Pulse pressure, mmHg	50.5 (11.2)	56.2 (16.1)	1.86	0.179	${\text{η}}_{p}^{2}$ = 0.038
Current smoker (%)	5.3	9.1	0.22	0.641	φ_c_ = 0.067
History of cardiovascular disease (%)	5.3	9.1	0.22	0.641	φ_c_ = 0.067
History of hypertension (%)	52.6	100.0	8.24	0.004	φ_c_ = 0.410
History of atrial fibrillation (%)	10.5	9.1	0.02	0.890	φ_c_ = 0.020
GDS	3.6 (3.9)	6.1 (3.7)	3.31	0.075	${\text{η}}_{p}^{2}$ = 0.069
**Cognitive measures^∗∗∗^**					
Mean (SD)					
Global cognition (DRS total score)	140.5 (3.4)	138.3 (4.5)	0.94	0.338	${\text{η}}_{p}^{2}$ = 0.021
Memory composite	0.18 (0.8)	-0.78 (0.9)	5.80	0.020	${\text{η}}_{p}^{2}$ = 0.117
Executive function/processing speed composite	0.20 (0.7)	-0.83 (1.1)	11.72	0.001	${\text{η}}_{p}^{2}$ = 0.218

*T2DM, type 2 diabetes mellitus; SD, standard deviation; FSRP, Framingham stroke risk profile (D’Agostino et al., 1994); GDS, geriatric depression scale; DRS, Mattis dementia rating scale (Mattis, 1988).*

*^∗^Data are mean and standard deviation in parentheses unless otherwise noted.*

*^∗∗^Modified FSRP omitting diabetes.*

*^∗∗∗^DRS total score is a raw score (maximum possible score = 144); memory and executive function/processing speed composite scores are *z* scores. Results for the group comparisons on DRS and memory and executive function/processing speed composite scores are from analysis of covariance (ANCOVA) models adjusted for age, sex, and education.*

### Regional CBF by T2DM

**FIGURE 2:**
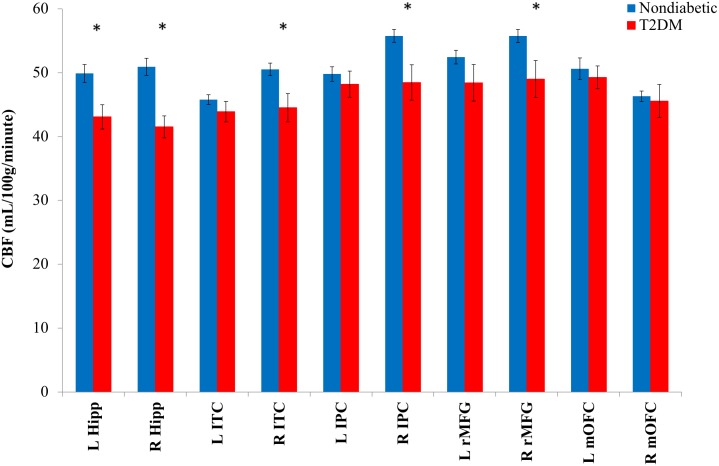
Mean regional CBF for T2DM and nondiabetic control groups. CBF, cerebral blood flow; L, left; R, right; hipp, hippocampus; ITC, inferior temporal cortex; IPC, inferior parietal cortex; rMFG, rostral middle frontal gyrus; mOFC, medial orbitofrontal cortex. ^∗^*p* < 0.05. Error bars represent ± 1 standard error.

Group means and differences in CBF for *a priori* ROIs are shown in **Figure 2**. MANCOVA adjusting for age, sex, aggregate vascular risk (i.e., modified FSRP omitting diabetes), and reference CBF (postcentral gyrus) revealed significant effects of T2DM on regional CBF. Specifically, compared to nondiabetic control participants, individuals with T2DM exhibited lower CBF in left hippocampus [*F*(1,43) = 5.45, *p* = 0.024, ${\text{η}}_{p}^{2}$ = 0.113], right hippocampus [*F*(1,43) = 8.81, *p* = 0.005, ${\text{η}}_{p}^{2}$ = 0.170], right inferior parietal cortex [*F*(1,43) = 11.78, *p* = 0.001, ${\text{η}}_{p}^{2}$ = 0.215], right inferior temporal cortex [*F*(1,43) = 8.00, *p* = 0.007, ${\text{η}}_{p}^{2}$ = 0.157], and right rostral middle frontal gyrus [*F*(1,43) = 9.38, *p* = 0.004, ${\text{η}}_{p}^{2}$ = 0.179]. Statistical significance of all results described above was retained using a 0.05 FDR.

In contrast, there were no significant group differences for left inferior parietal cortex [*F*(1,43) = 0.01, *p* = 0.906, ${\text{η}}_{p}^{2}$ < 0.001], left inferior temporal cortex [*F*(1,43) = 2.01, *p* = 0.163, ${\text{η}}_{p}^{2}$ = 0.045], left medial orbitofrontal cortex [*F*(1,43) = 0.16, *p* = 0.690, ${\text{η}}_{p}^{2}$ = 0.004], right medial orbitofrontal cortex [*F*(1,43) = 0.02, *p* = 0.903, ${\text{η}}_{p}^{2}$ < 0.001], or left rostral middle frontal gyrus [*F*(1,43) = 1.88, *p* = 0.178, ${\text{η}}_{p}^{2}$ = 0.042]. Also, as expected, we confirmed that there were no group differences in terms of postcentral gyrus reference CBF [*F*(1,47) = 0.78, *p* = 0.382, ${\text{η}}_{p}^{2}$ = 0.016].

### Regional Cortical Thickness and Volume by T2DM

Multiple analysis of covariance models adjusting for age, sex, and aggregate vascular risk (i.e., modified FSRP omitting diabetes) (and intracranial volume for analyses with hippocampal volume and WM hypointensities as the dependent variable) examined differences in cortical thickness or volume for the *a priori* ROIs used in the CBF analyses as well as for WM hypointensities. These models revealed no significant group differences in terms of cortical thickness of the right or left inferior parietal cortices [left: (*F*(1,44) = 0.58, *p* = 0.452, ${\text{η}}_{p}^{2}$ = 0.013); right: (*F*(1,44) = 0.50, *p* = 0.502, ${\text{η}}_{p}^{2}$ = 0.010)], inferior temporal cortices [left: (*F*(1,44) = 0.27, *p* = 0.607, ${\text{η}}_{p}^{2}$ = 0.006); right: (*F*(1,44) = 0.17, *p* = 0.687, ${\text{η}}_{p}^{2}$ = 0.004)], medial orbitofrontal cortices [left: (*F*(1,44) = 0.003, *p* = 0.955, ${\text{η}}_{p}^{2}$ < 0.001); right: (*F*(1,44) = 0.37, *p* = 0.545, ${\text{η}}_{p}^{2}$ = 0.008)], or rostral middle frontal gyri [left: (*F*(1,44) = 1.44, *p* = 0.237, ${\text{η}}_{p}^{2}$ = 0.032); right: (*F*(1,44) = 0.94, *p* = 0.337, ${\text{η}}_{p}^{2}$ = 0.021)]. Similarly, there were no significant differences between those with T2DM and the nondiabetic control participants in left or right hippocampal volume [left: (*F*(1,43) = 1.32, *p* = 0.258, ${\text{η}}_{p}^{2}$ = 0.030); right: (*F*(1,43) = 2.06, *p* = 0.158, ${\text{η}}_{p}^{2}$ = 0.046)] or volume of WM hypointensities [*F*(1,43) = 0.83, *p* = 0.367, ${\text{η}}_{p}^{2}$ = 0.019].

### Associations Between Regional CBF and Cognition

Correlation analyses were used to examine associations between CBF (in regions for which group differences were found by T2DM status) and cognitive abilities subserved by those particular regions. That is, analyses were performed relating bilateral hippocampal and right inferior temporal CBF to memory and right inferior parietal and right rostral middle frontal CBF to executive function/processing speed. Associations were examined both unadjusted and controlling for diabetes status (present versus absent).

**FIGURE 3:**
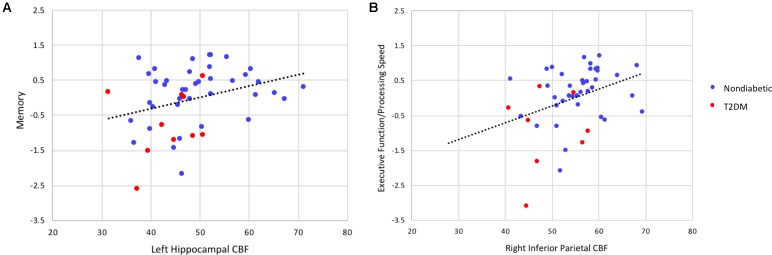
Scatterplots of correlations between cognitive composite scores (*z*-scores) and regional cerebral blood flow (in units of milliliters per 100 g of tissue per minute). **(A)** Depicts the correlation between memory performance and left hippocampal CBF and **(B)** the correlation between executive function/processing speed and inferior parietal CBF (all *p*’s < 0.05). These significant associations were maintained controlling the FDR at 0.05. When partial correlation analyses were performed adjusting for diabetes status, the association between executive function/processing speed and regional CBF remained statistically significant (*p* < 0.05) whereas the association between memory and regional CBF was no longer significant.

Collapsed across the entire sample, there were significant associations between lower regional CBF and poorer cognitive performance (see **Figure 3** for selected associations for memory and executive function/processing speed with regional CBF). Lower hippocampal and inferior temporal cortex CBF was associated with poorer memory performance. Significant CBF-memory correlations were found for both left hippocampal CBF (*r* = 0.31, *p* = 0.016) and right hippocampal CBF (*r* = 0.25, *p* = 0.044). In contrast, lower right inferior temporal CBF was not significantly associated with poorer memory performance (*r* = 0.07, *p* = 0.310). Significant associations were maintained when FDR was limited to 0.10. In contrast, the association with memory and left hippocampal CBF but not right hippocampal CBF remained significant when FDR was limited to 0.05.

When partial correlations adjusted for diabetes status were performed, the associations between regional CBF and memory were attenuated and no longer statistically significant (left hippocampus: *r* = 0.15, *p* = 0.168; right hippocampus: *r* = 0.02, *p* = 0.458; right inferior temporal gyrus: *r* = -0.10, *p* = 0.253).

Lower CBF in right inferior parietal cortex and rostral middle frontal gyrus was associated with poorer performance on measures of executive function/processing speed (right inferior parietal cortex: *r* = 0.36, *p* = 0.007; right rostral middle frontal gyrus: *r* = 0.34, *p* = 0.009). These significant associations were maintained when FDR was limited to 0.05.

When partial correlation analyses were performed adjusting for diabetes status, the associations between regional CBF and executive function/processing speed were somewhat attenuated although they remained statistically significant for both the right inferior parietal cortex (*r* = 0.26, *p* = 0.043) and right rostral middle frontal gyrus (*r* = 0.29, *p* = 0.028).

### Sensitivity Analyses

Given the higher proportion of men in the T2DM group, we performed sensitivity analyses to examine the potential role of sex on CBF in our sample. Similar to our primary analyses examining CBF by T2DM status, we ran MANCOVA models adjusting for age, aggregate vascular risk (i.e., modified FSRP omitting diabetes), and reference CBF (postcentral gyrus) to assess sex-related differences in CBF for those regions where group differences were observed between the nondiabetic controls and T2DM participants. The MANCOVA models revealed no significant differences between men and women across any of these ROIs [left hippocampus: (*F*(1,44) = 0.04, *p* = 0.848, ${\text{η}}_{p}^{2}$ = 0.001); right hippocampus: (*F*(1,44) = 0.54, *p* = 0.465, ${\text{η}}_{p}^{2}$ = 0.012); right inferior parietal: (*F*(1,44) = 1.62, *p* = 0.210, ${\text{η}}_{p}^{2}$ = 0.035); right inferior temporal: (*F*(1,44) = 0.75, *p* = 0.393, ${\text{η}}_{p}^{2}$ = 0.017); right rostral middle frontal: (*F*(1,44) = 0.57, *p* = 0.454, ${\text{η}}_{p}^{2}$ = 0.013)].

In addition, we also performed *t*-test analyses to compare a subset of the sample consisting of nondiabetic control (*n* = 11) and T2DM (*n* = 11) participants who were matched so that the two groups did not significantly differ in terms of demographic or covariate variables including age; sex; aggregate vascular risk; pulse pressure, systolic blood pressure, or diastolic blood pressure; history of cardiovascular disease or atrial fibrillation; or depression (i.e., GDS score). In this matched subsample, findings for group differences in CBF remained statistically and qualitatively similar to the results from analyses including the entire sample. That is, when the T2DM and nondiabetic control groups were equivalent in terms of sex distribution and vascular risk covariates, the T2DM participants showed reduced CBF in left hippocampus (*t* = 3.29, *p* = 0.004, Cohen’s *d* = 1.40), right hippocampus (*t* = 4.10, *p* = 0.001, Cohen’s *d* = 1.75), right inferior parietal cortex (*t* = 2.68, *p* = 0.014, Cohen’s *d* = 1.14), right inferior temporal cortex (*t* = 2.90, *p* = 0.009, Cohen’s *d* = 1.24), and right rostral middle frontal gyrus (*t* = 2.85, *p* = 0.104, Cohen’s *d* = 1.21). Similar to the primary analyses including the entire sample, there were no significant group differences in any of the other *a priori* ROIs (left inferior parietal cortex: *t* = 0.95, *p* = 0.352, Cohen’s *d* = 0.41; left inferior temporal cortex: *t* = 1.63, *p* = 0.118, Cohen’s *d* = 0.70; left medial orbitofrontal cortex *t* = 1.21, *p* = 0.240, Cohen’s *d* = 0.52; right medial orbitofrontal cortex: *t* = 0.75, *p* = 0.462, Cohen’s *d* = 0.32; left rostral middle frontal cortex: *t* = 1.74, *p* = 0.097, Cohen’s *d* = 0.74).

## Discussion

Our results demonstrate that CBF is reduced in nondemented older adults with T2DM independent of age, sex, and related vascular risk factors. We did not find significant differences between those with and without T2DM in terms of brain structure (cortical thickness or brain volume in regions of interest), suggesting that CBF alterations occur independent of cerebral atrophy and may precede structural changes that have been identified in previous studies. T2DM-related reductions in CBF were pronounced in known predilection sites for AD pathology as well as regions implicated in cerebrovascular dysfunction in early AD (hippocampus, inferior parietal cortex, inferior temporal cortex, and middle frontal regions). Moreover, among older adults both with and without T2DM, lower CBF was associated with poorer cognitive performance in memory and executive/processing speed domains. These findings were somewhat attenuated when analyses were adjusted for diabetes status although associations between regional CBF and executive function/processing speed remained significant in adjusted analyses. Findings add to a growing body of research suggesting that perfusion deficits may underlie cognitive decrements frequently observed among older adults with T2DM. Results also suggest that CBF measured with ASL MRI may reflect an early and important marker of risk of cognitive decline in T2DM and related conditions particularly given that the mean level of performance on cognitive measures in our sample was within the normal range and not objectively impaired.

Several previous studies have examined the association of T2DM and CBF using techniques including PET, SPECT, and ASL, although findings across studies have been mixed. Many studies have reported reduced CBF in T2DM (Last et al., 2007; Xia et al., 2015; Cui et al., 2017; Dai et al., 2017), although some other studies have reported no differences between individuals with and without T2DM (Tiehuis et al., 2008; Launer et al., 2015; Rusinek et al., 2015). As noted by Dai et al. (2017), studies reporting no alterations in CBF in T2DM relative to nondiabetic control participants typically examined large ROIs such as whole brain GM or large cortical regions. Importantly, previous studies are limited in their ability to reliably detect CBF reductions in the context of T2DM as they typically did not consider the effects of additional vascular risk factors such as hypertension (Dai et al., 2017) or elevated pulse pressure, which commonly co-occur with T2DM, or brain atrophy, which in some instances appears to fully account for decreased CBF (Sabri et al., 2000). Furthermore, few studies have utilized a PCASL sequence and implemented an ASL protocol optimized for use in older adults.

Despite previous findings linking T2DM and elevated blood glucose to cognitive impairment and AD, there are few studies investigating the neuropathologic mechanisms underlying these associations. Although autopsy-based studies have shown that T2DM is linked to cerebral infarcts (Peila et al., 2002; Arvanitakis et al., 2006; Sarwar et al., 2010), its association with AD neuropathology itself (i.e., β-amyloid plaques, Aβ and neurofibrillary tangles, NFT) remains unclear. (Peila et al., 2002; Beeri et al., 2005; Arvanitakis et al., 2006; Ahtiluoto et al., 2010; Malek-Ahmadi et al., 2013). However, some evidence suggests a role for deficiencies in brain insulin in the pathogenesis of AD and have proposed that AD may be “type 3 diabetes” (Steen et al., 2005). In our previous work, we found that midlife elevated blood glucose is predictive of more severe AD pathology (i.e., higher medial temporal lobe NFT pathology) in late life. This work suggests that elevated blood glucose – even many years before death and even among nondiabetic individuals – may have detrimental effects on the brain that ultimately contribute to the development of AD pathology and subsequent cognitive decline (Bangen et al., 2016). The present findings provide additional evidence for the influence of T2DM on changes in AD-vulnerable regions, and they suggest that cerebrovascular dysfunction may underlie the predilection of AD pathology in these regions.

We found lower CBF in T2DM in *a priori* ROIs including medial temporal lobe, parietal, and frontal regions. This pattern is similar to that seen in AD and vascular disease. Medial temporal regions are susceptible to early pathologic and neurodegenerative changes in AD, and alterations in CBF represent a potential mechanism through which these changes may occur. Indeed, many of the regions implicated in the current study, including inferior parietal cortices, are key components of the default mode network, which contributes to episodic memory and executive functioning and has been implicated in preclinical AD (Hampel, 2013). Lifetime cerebral metabolism associated with default mode network activity may predispose these regions to AD-related pathologic changes including Aβ accumulation and may also disrupt connections with the medial temporal lobe, resulting in impaired cognitive function (Buckner et al., 2005). Further, WM lesion pathology in the parietal lobe has been implicated as an early biomarker of AD, and as a marker of small-vessel disease, these lesions may reflect later-stage consequences of chronic hypoperfusion in this region (Lee et al., 2016).

The present findings corroborate previous studies demonstrating hypoperfusion in T2DM in the absence of brain volume differences, suggesting that perfusion alterations are independent of cerebral atrophy (Xia et al., 2015; Jansen et al., 2016). Previous studies have shown that functional changes precede structural changes in the context of AD (Devous, 2002) and T2DM (Musen et al., 2012). Given this, MRI techniques such as ASL have great potential as a non-invasive method for detecting early and/or subtle functional brain changes in asymptomatic individuals. Advanced MRI techniques that measure early physiological changes including alterations in CBF may elucidate the mechanisms that precede the development of irreversible parenchymal/structural damage and serve as a marker of risk for cognitive decline.

Indeed, mounting evidence suggests that ASL CBF represents a useful biomarker in at-risk individuals given that this technique can reliably differentiate those at risk from control participants (Fleisher et al., 2009; Bangen et al., 2012; Wierenga et al., 2012). Furthermore, longitudinal studies have shown that ASL CBF indices predict cognitive decline in older adults with normal cognition (Xekardaki et al., 2015) as well as progression from normal cognition to MCI (Beason-Held et al., 2013), and MCI to AD (Chao et al., 2010). Previous work has shown that CBF alterations are independent of changes in volume and were detectable several years prior to the development of cognitive impairment (Beason-Held et al., 2013). Our current findings emphasize the important link between CBF and cognition, and they provide further support for CBF as a useful marker of vascular risk and correlate of cognitive functioning in nondemented older adults. Furthermore, a recent study showed that there was an increase in perfusion and improvements in cognitive performance after insulin administration in individuals with T2DM which was greater than in the nondiabetic control group, and these insulin-induced changes were associated with vasodilation in the middle cerebral artery territory, suggesting involvement of a vascular mechanism (Novak et al., 2014). Although findings have been mixed, overall it appears that reduced CBF seems to be an early change independent of brain structural changes and may be a viable intervention target for preventing cognitive decline in T2DM. Dissemination of methods capable of detecting cerebrovascular dysfunction prior to the manifestation of these frank lesions would represent a major advancement in early detection and expansion of treatment opportunities to prevent or delay cognitive impairment in these individuals.

Taken together, our findings show an important relationship between cerebral perfusion and memory and executive function in the context of T2DM. Results further highlight the potential value in examining ASL CBF as a sensitive vascular marker in aging, metabolic and vascular conditions, and dementia risk. Strengths of this work include a well-characterized sample, comprehensive neuropsychological assessment, and use of an ASL protocol optimized for use in older adults. However, there are important limitations of this study worth noting. First, our sample size of participants with T2DM is small and thus results should be considered preliminary. Data on glycemic control was not available (e.g., hemoglobin A1c) and therefore not included in our analyses, which should be addressed in future studies. In addition, our sample was predominantly white, generally medically healthy, and relatively well-educated, which may affect generalizability of the findings. Future studies should include larger sample sizes and longitudinal follow-up spanning middle age to older age in order to better understand how changes in CBF may evolve over time and how they influence the development of later-stage structural and pathologic changes that have previously been associated with T2DM. Finally, the nondiabetic control group included a higher proportion of women relative to the T2DM group. Although women have been shown to have higher CBF, some evidence suggests that this difference is diminished with advancing age and that, by the sixth decade, men and women show similar CBF rates (Gur et al., 1987; Gur and Gur, 1990). The mean age of the present sample was approximately 73 (range = 68–88). We performed sensitivity analyses to examine the potential role of sex on CBF in our sample and found that sex was not significantly associated with regional CBF. We performed a second set of sensitivity analyses in a subset of our sample including nondiabetic control participants and those with T2DM who were matched on distribution of sex, pulse pressure and blood pressure, and other important demographic and vascular risk variables. Findings were qualitatively and statistically similar to those from the primary analyses including the entire sample. Nonetheless, future work should aim to replicate our findings in a larger sample with groups matched on sex distribution.

Despite these limitations, our findings, if replicated, may have important research and clinical implications. Indeed, our results show that reduced CBF may be an early marker of incipient change independent of brain structural changes, and it may be a viable intervention target for preventing cognitive decline in T2DM. Dissemination of methods capable of detecting cerebrovascular dysfunction prior to the manifestation of these frank lesions would represent a major advancement in early detection and expansion of treatment opportunities to prevent or delay cognitive impairment in these individuals. For example, pharmacological and behavioral interventions, such as physical exercise, may influence the regulation of CBF and, ultimately, the prevention of cognitive decline in T2DM and related conditions. T2DM is a growing condition that has been linked to the development of substantial cognitive and brain changes, and there is therefore a pressing public health need to identify early biomarkers of cognitive decline in T2DM and related conditions as well as potentially modifiable mechanisms underlying these changes. Such research will help facilitate the development and optimization of targeted interventions to reduce dementia risk while improving the health and functioning of individuals with T2DM and other groups at risk for cognitive decline.

## Author Contributions

KB designed the study, analyzed and interpreted the data, and wrote and revised the manuscript. MW wrote and revised the manuscript. AW, NE, and AC created figures and revised the manuscript for important intellectual content. EE, LD-W, KT, DN, TL, and MB interpreted the data and revised the manuscript for important intellectual content. All authors approved the submitted version of the manuscript and agree to be accountable for all aspects of the work.

## Conflict of Interest Statement

The authors declare that the research was conducted in the absence of any commercial or financial relationships that could be construed as a potential conflict of interest.

## Footnotes

1 cbfbirn.ucsd.edu
